# A Fiber Bragg Grating—Bimetal Temperature Sensor for Solar Panel Inverters

**DOI:** 10.3390/s110908665

**Published:** 2011-09-06

**Authors:** Mohd Afiq Ismail, Nizam Tamchek, Muhammad Rosdi Abu Hassan, Katrina D. Dambul, Jeyrai Selvaraj, Nasrudin Abd Rahim, Reza Sandoghchi, Faisal Rafiq Mahamd Adikan

**Affiliations:** 1 Department of Electrical Engineering, Faculty of Engineering, University of Malaya, 50603 Kuala Lumpur, Malaysia; E-Mails: nizamtm@gmail.com (N.T.); muhdrosdi22@gmail.com (M.R.A.H.); katrina@mmu.edu.my (K.D.D.), jeyraj@um.edu.my (J.S.); nasrudin@um.edu.my (N.A.R.); rezasandoghchi@gmail.com (R.S.); rafiq@um.edu.my (F.R.M.A.); 2 Faculty of Engineering, Multimedia University, 63100 Cyberjaya, Selangor, Malaysia; 3 Photonic Research Centre, University of Malaya, 50603 Kuala Lumpur, Malaysia

**Keywords:** Fiber Bragg Grating, bimetal strip, temperature sensor, solar panel inverter

## Abstract

This paper reports the design, characterization and implementation of a Fiber Bragg Grating (FBG)-based temperature sensor for an Insulted-Gate Bipolar Transistor (IGBT) in a solar panel inverter. The FBG is bonded to the higher Coefficient of Thermal Expansion (CTE) side of a bimetallic strip to increase its sensitivity. Characterization results show a linear relationship between increasing temperature and the wavelength shift. It is found that the sensitivity of the sensor can be categorized into three characterization temperature regions between 26 °C and 90 °C. The region from 41 °C to 90 °C shows the highest sensitivity, with a value of 14 pm/°C. A new empirical model that considers both temperature and strain effects has been developed for the sensor. Finally, the FBG-bimetal temperature sensor is placed in a solar panel inverter and results confirm that it can be used for real-time monitoring of the IGBT temperature.

## 1. Introduction

Solar panel inverters are used to change direct current (DC) from solar panels into alternate current (AC). The operating range of a solar panel inverter is from 2.0 kW to 7.5 kW. The change from DC to AC is achieved via an electrical switching process. Most inverters use the Pulse Width Modulation (PWM) technique to reduce harmonics [1,2]. A typical implementation of IGBTs in a solar panel inverter uses full-bridge topology using four switches. However, solar panel inverters that use this technique emit large EMI due to high *di/dt* and *dv/dt* caused by switching [3]. Furthermore, because of its semiconductor foundation, the temperature variations can cause the IGBT characteristics to change dramatically. As the temperature increases, the turn-off energy losses increase and forward voltage drop, *V_CE_*, decreases [4].

Electronic-based temperature sensor measurement of an IGBT is impractical because of the large EMI presence in the solar panel inverter. High EMI levels can cause disturbances in the temperature readings. Therefore, an alternative option is to use FBG-based sensors, because of their immunity to EMI. Fiber Bragg Gratings (FBG) have been traditionally used as temperature, strain and vibration sensors [5–7]. Variations in temperature, strain and vibration induce changes in the grating period and index of refraction of the fiber optic, thus changing the Bragg wavelength. FBGs offer important advantages such as electrically passivity, immunity to electromagnetic interference (EMI), high sensitivity, multiplexing and self-referencing capability. Immunity to EMI makes FBGs an alternative sensor when EMI can cause disturbances to electronic-based sensors. FBGs are utilized as temperature sensors in electric power systems where high voltage is a concern [8].

The dependence of the Bragg wavelength on temperature effect is due to two factors: (i) the dependence of the index of refraction of the glass to temperature and (ii) thermal expansion of the glass. In silica fibers, ∼95% of the observed shift in the Bragg wavelength occurs due to shift in the fiber’s refractive index with respect to changes in the temperature. A bare FBG temperature sensor with a wavelength of 1.3 μm over the range of 5 °C to 85 °C gives a normalized responsivity of 6.67 × 10^−6^ °C^−1^ [9]. A typical value for thermal response at 1,550 nm is 0.01 nm/°C. At higher temperature the sensitivity is higher and the response becomes slightly nonlinear [10]. An FBG is essentially not very sensitive to the change of the external refractive index (RI) [11]. By attaching the FBG to passive devices, the thermal characteristics of the materials can be used to control the sensitivity of the FBG wavelength to temperature.

Reddy *et al.* [12] made use of the chemical composition of the gratings and attached an FBG to a platinum plate in order to use the FBG to measure high temperatures (up to 900 °C). The sensor gives an experimental sensitivity value of 11.44 pm/°C. Wu *et al.* [13] used capillary steel tubes, metalized and organic polymer packages to enhance the sensitivity of an FBG for measurements of low-temperatures. The temperature sensitivities of the capillary steel tube, metalized and organic polymer packages were 0.0213 nm/°C, 0.0283 nm/°C and 0.1376 nm/°C, respectively.

In general, the temperature sensitivity of the FBG can be improved when it is coated with a material that has high CTE, such as a metal. The metal coating also protects the FBG. Feng *et al.* [14] coated a FBG with Ni-Cu, Cu-Ni and Cu. Lupi *et al.* [15] coated the FBG with zinc and copper using the traditional electrowinning process, after an aluminum pre-coating of the sensor. However, if an abrupt thermal stress (occurring at much lower temperature) occurs during the coating process, this can permanently affect the optical features of the FBG and the optical properties will also be slightly affected by the coating process.

Tian [16] and Song [17] bonded FBGs to the lower CTE side of a bimetal and used it as a sensor to measure temperature and strain. In this work, we propose to improve the sensor’s sensitivity by bonding the FBG to the higher CTE side of a bimetal. The FBG-Bimetal temperature sensor is first characterized in order to understand its behavior. Although the FBG-Bimetal temperature sensor is affected by the strain and temperature effects simultaneously, we are able to separate the two components in order to determine the temperature of the IGBT.

We have developed a temperature measuring system that is simple, cheap, effective and can be fully integrated into a solar panel inverter. Packaging or coating the FBG with high CTE metals is expensive and time-consuming. It would also be difficult to integrate into a solar panel inverter to measure the temperature of the IGBT. By removing the middle rivet of the bimetallic sheet, the FBG-Bimetal temperature sensor can be screwed on top of the IGBT. To the best of the authors’ knowledge, this is the first paper that describes an FBG-based temperature sensor for solar panel inverters.

## 2. Methodology

### Theory and Design

The Bragg wavelength is described by:

(1) $$\lambda_{B}=2n_{\mathrm{eff}}\Lambda$$

From Equation (1), it is apparent that the Bragg wavelength, *λ_B_*, is depended on the effective index of refraction, *n_eff_* and the spacing between gratings, Λ. The effect of temperature to the Bragg wavelength under constant strain is dominated by the thermo-optic effect, which accounts for 95% of the total effect. The wavelength shift due to temperature effect on an FBG is given by [10]:

(2) $$\frac{{\delta \lambda }_{B}}{\lambda_{B}}=\alpha +\frac{1}{n}\frac{\mathrm{dn}}{\mathrm{dT}}$$

where *α* is the Coefficient of Thermal Expansion (CTE) of the fiber material (e.g., silica). The strain effect on wavelength shift is given by [10]:

(3) $$\frac{{\delta \lambda }_{B}}{\lambda_{B}}=[1-p_{e}]ɛ$$

where the photoelastic contribution, *p_e_*, is given by [10]:

(4) $$p_{e}=(n^{2}/2)[p_{12}-\mu (p_{11}+p_{12})]$$

where *p_ij_* is the fiber Pockel’s coefficient and *μ* is the Poisson ratio.

The FBG is bonded to a bimetal, so when it is heated, the wavelength shift is the product of strain effect, Δ*ε* and temperature variation, Δ*T*. Therefore, the wavelength shift of the FBG is given by [6]:

(5) $$\frac{{\Delta \lambda }_{B}}{\lambda_{B}}=K_{ɛ}\Delta ɛ+K_{T}\Delta T$$

where *K_ε_* and *K_T_* are the strain and temperature sensitivities of the FBG, respectively.

A bimetallic sheet consists of two metals with different CTE. When there is a change in the temperature, both metals expand in a pre-determined manner due to their CTE differences. When heated, the metal with the higher CTE will expand more than the metal with the lower CTE. As a result, the bimetallic sheet will bend towards the metal with the lower CTE. When cooled, the condition is reversed and consequently, the bi imetallic sheet will bend in the opposite direction.

The strain, Δ*ε*, that influences the wavelength shift in Equation (5), is the strain of the bimetallic sheet when the temperature varies. Therefore, we associate the function Δ*ε* with the temperature variation, Δ*T*. The relationship between the strain, *ε*, of the bimetallic sheet and the temperature variation, Δ*T*, is given [18] by:

(6) Δɛ=ΔαΔT

where Δ*α* is the difference of the coefficient of thermal expansion (CTE) of the two metals.

Therefore, the wavelength shift for an FBG that is bonded to a bimetal can be rewritten as:

(7) $$\frac{{\Delta \lambda }_{B}}{\lambda_{B}}=K_{ɛ}{\Delta \alpha \Delta T}_{\text{bimetal}}+K_{T}{\Delta T}_{\text{FBG}}$$

In Equation (7), there are two temperature variations, namely, Δ*T*_bimetal_ and Δ*T*_FBG_ due to the heat transfer loss between the FBG and the bimetallic sheet. Therefore, the value of Δ*T*_bimetal_ is not the same as Δ*T*_FBG_ although the heat is generated from the same source. Also, from Equation (7), it is understood that the relationship between wavelength shift and temperature is linear.

## 3. Experimental Setup

**Figure 1.**
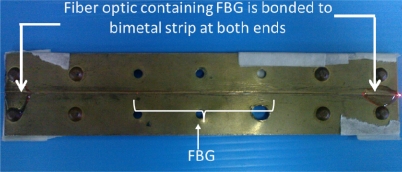
FBG-Bimetal temperature sensor assembly.

The polyimide coating of the FBG is removed before the FBG is inscribed using a 244 nm ultraviolet (UV) laser exposure with a phase mask, using hydrogen-loaded fibers. The peak reflectivity values are typically 90% and the Bragg wavelength located around 1,550 nm at room temperature. The physical grating lengths are set to 2 cm length for all samples. A bimetallic sheet measuring 150 mm (height) × 32 mm (width) × 8.4 mm (thickness) is used. The FBG is glued on the bimetallic sheet using UV cured Norland Optical Adhesive (number 61). The glue has a melting temperature of 125 °C. The bimetallic sheet consists of brass and steel riveted together. The FBG was bonded onto the brass surface, which has the higher CTE. Figure 1 shows the FBG-Bimetal temperature sensor assembly.

### Calibration of Sensor

**Figure 2.**
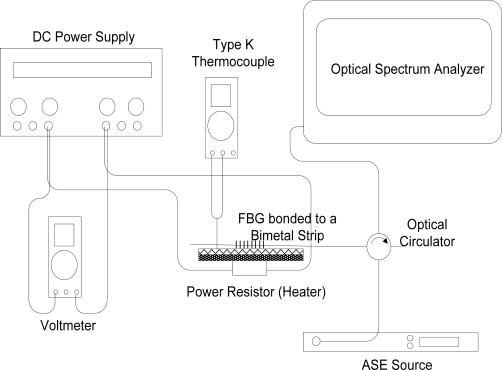
Experimental setup for the sensor calibration.

The experimental setup for the calibration of the temperature sensor is shown in Figure 2. When heated, brass elongates more than steel. As a result, the bimetallic sheet curves towards steel. The periodicity of the FBG grating increases because the FBG is stretched. With increasing temperature, the Bragg wavelength, *λ_B_*, will continue to increase.

A DC power source controls the voltage supply to a 50 W, 100 Ω power resistor which functions as a heater. The voltmeter monitors the voltage supply. Type K thermocouple temperature sensor was used to monitor the temperature of the bimetallic sheet. Amplified Spontaneous Emission (ASE) source (NP2000ASE, Nuphoton Technologies) supply C-band light into the FBG and the reflected Bragg wavelengths are monitored by an optical spectrum analyzer (FTB-5240S/BP Optical Spectrum Analyzers, EXFO). The voltage of the DC power source is gradually increased in order to increase the temperature of the bimetallic sheet. The corresponding Bragg wavelength for each temperature increment is recorded. The wavelength shift of the FBG mounted onto the bimetal is characterized with respect to the temperature changes and the elongation of bimetal. Repeat measurements are taken to account for various errors.

## 4. Result and Discussion

**Figure 3.**
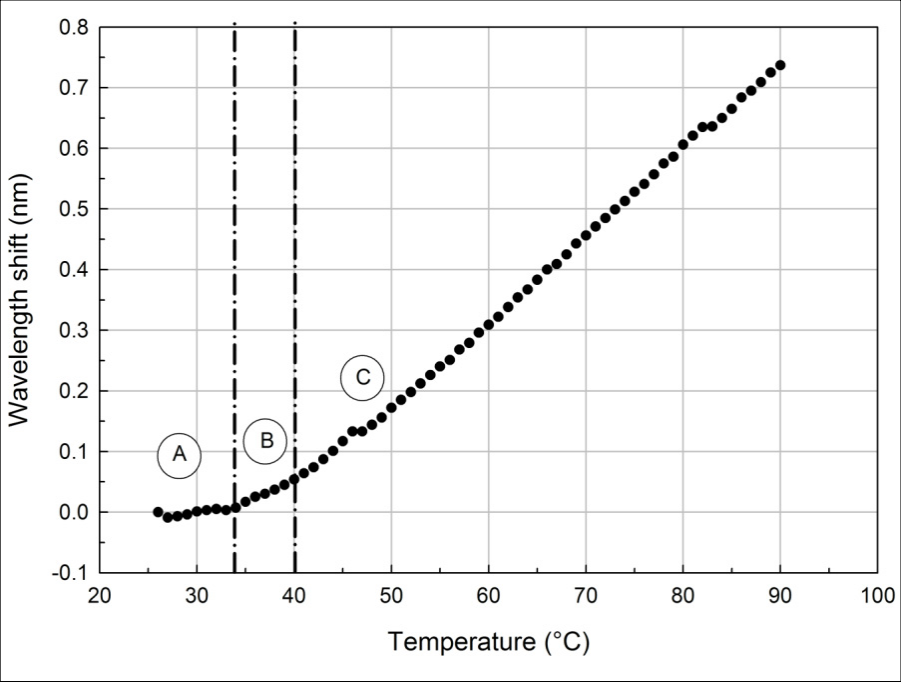
Wavelength shift *versus* temperature.

The result of the characterization process is shown in Figure 3. As the temperature increases, the wavelength shift also increases. Figure 3 shows that three different regions have been identified and are labeled as A, B and C, respectively.

In region A, from 26 °C to 34 °C, the gradient is 1 pm/°C. In region B, from 35 °C to 40 °C, the gradient is 7 pm/°C and in region C, from 41 °C to 90 °C the gradient is 14 pm/°C. Tian [16] reported a temperature sensitivity of −4 pm/°C, while Song [17] reported a temperature sensitivity of 8.1 pm/°C and −0.018 pm/°C for uncompensated and compensated FBGs, respectively. Compared to the work by Tian [16] and Song [17], the FBG-Bimetal temperature sensor is more sensitive in region C.

This concurs with Equation (7), where the relationship between wavelength shift and strain and temperature is linear. The results also show that the FBG-Bimetal temperature sensor is able to meet all requirements without any modifications. From the calculation based on the expressions presented in Equations (5–7) the value of Δ*T_FBG_* in region C is 0.73 °C when Δ*T_bimetal_* is 1 °C. This indicates that there is 27% of heat transfer loss between the bimetal and the FBG. Consequently, Δ*T_FBG_* can be written as 0.73 Δ*T_bimetal_*. Thus, Equation (7) becomes:

(8) $$\frac{{\Delta \lambda }_{B}}{\lambda_{B}}=K_{ɛ}{\Delta \alpha \Delta T}_{\text{bimetal}}+K_{T}(0.73){\Delta T}_{\text{bimetal}}$$

### Real-Time Monitoring IGBT Temperature

**Figure 4.**
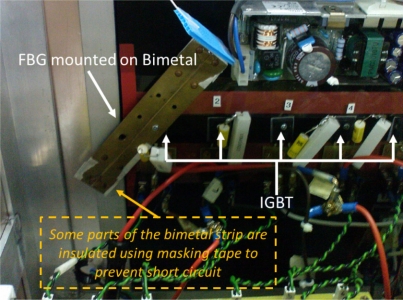
FBG-Bimetal temperature sensor screwed on top of an IGBT.

Following characterization, the FBG-Bimetal temperature sensor is placed inside a solar panel inverter for real-time testing. The sensor is screwed on to the top of an IGBT as depicted in Figure 4. The IGBT is facing the steel side and the FBG is bonded onto the brass side of the bimetallic sheet. Therefore, the FBG is not under any additional strain from the mounting process. The FBG wavelength shift is continuously monitored every 5 min from 10:30 a.m. to 6 p.m. The Bragg wavelength of this sensor system is then recorded and processed using the *Labview* software package.

**Figure 5.**
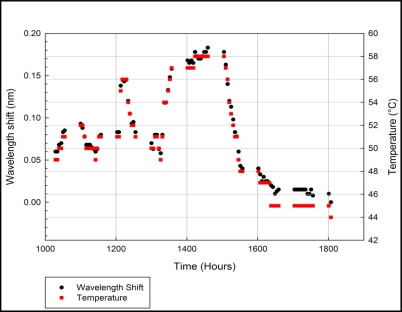
IGBT temperature measurements using FBG-Bimetal temperature sensor.

Figure 5 shows the functionality of the FBG-Bimetal temperature sensor when it is placed in a solar panel inverter. The real-time monitoring process is performed from 10:30 a.m. to 6 p.m. with 5 min intervals between the temperature measurements. The temperature measurements are performed automatically via a previously developed *Labview* software program. The test was performed on a cloudy day.

The IGBT temperature fluctuates according to the solar power collected by the solar panel. The power collected from the solar panel depended on the position of the sun during the day or whether the day is overcast. Therefore, the temperature of the IGBT should gradually increase in the morning, peak in the afternoon and gradually decrease in the evening. From Figure 5, the temperature of IGBT was high around 12 p.m. and around 2 p.m. The highest temperature measured was 58 °C from 2:30 p.m. to 3 p.m. The temperature of IGBT gradually decreased in the evening as the sun sets in. The results confirmed that the FBG-Bimetal temperature sensor is working correctly when placed in a solar panel inverter.

## 5. Conclusions

Characterization results show that there is a linear relationship between the wavelength shift and temperature changes. It is found that the sensitivity of the sensor can be categorized into three characterization temperature regions between 26 °C and 90 °C. The region from 41 °C to 90 °C shows the highest sensitivity, with a gradient of 14 pm/°C. As the temperature increases, the sensitivity increases and becomes slightly nonlinear. Therefore, we suspect that FBG itself has a temperature sensitivity threshold. Once the threshold is exceeded, the FBG temperature sensitivity increases. The wavelength shift in FBG-Bimetal temperature sensor is a product of the heat conduction between the two metallic elements and the FBG, and also the threshold condition of the FBG. When placed in a solar panel inverter, the FBG-Bimetal temperature sensor is able to detect the temperature of the IGBT.
